# Drug-Eluting Biodegradable Implants for the Sustained Release of *Bis*phosphonates

**DOI:** 10.3390/polym12122930

**Published:** 2020-12-07

**Authors:** Cintya Dharmayanti, Todd A. Gillam, Desmond B. Williams, Anton Blencowe

**Affiliations:** 1 Applied Chemistry and Translational Biomaterials Group, Clinical and Health Sciences, University of South Australia, Adelaide, SA 5000, Australia; Cintya.Dharmayanti@unisa.edu.au (C.D.); Todd.Gillam@unisa.edu.au (T.A.G.); 2 Surface Interactions and Soft Matter Group, Future Industries Institute, University of South Australia, Mawson Lakes, SA 5095, Australia; 3 Pharmacy and Biomedical Sciences, Clinical and Health Sciences, University of South Australia, Adelaide, SA 5000, Australia; Des.Williams@unisa.edu.au

**Keywords:** *bis*phosphonate, implant, osteoporosis, sustained release, poly(lactic acid) (PLA), poly(D,L-lactide-*co*-glycolide) (PLGA), hot-melt extrusion

## Abstract

Despite being one of the first-line treatments for osteoporosis, the *bis*phosphonate drug class exhibits an extremely low oral bioavailability (<1%) due to poor absorption from the gastrointestinal tract. To overcome this, and to explore the potential for sustained drug release, bioerodible poly(lactic acid) (PLA) and poly(D,L-lactide-co-glycolide) (PLGA) implants loaded with the *bis*phosphonate alendronate sodium (ALN) were prepared via hot-melt extrusion. The rate of drug release in vitro was modulated by tailoring the ratio of lactide to glycolide in the polymer and by altering the ALN-loading of the implants. All investigated implants exhibited sustained ALN release in vitro between 25 to 130 days, where implants of greater glycolide composition and higher ALN-loadings released ALN more rapidly. All PLGA implants demonstrated a sigmoidal release profile, characterised by an initial surface dissolution phase, followed by a period of zero-order drug diffusion, then relaxation or erosion of the polymer chains that caused accelerated release over the subsequent days. Contrastingly, the PLA implants demonstrated a logarithmic release profile, characterised by a gradual decrease in ALN release over time.

## 1. Introduction

Osteoporosis is a skeletal disorder characterised by bone fragility and increased susceptibility to fracture [1]. This occurs due to deterioration of bone microarchitecture and low bone mass caused by excessive osteoclastic bone resorption [2,3]. Osteoporosis is estimated to affect over 200 million individuals across the globe, with an estimated annual cost of $131.5 billion worldwide [3,4,5]. The first-line treatment for various subtypes of osteoporosis is the *bis*phosphonate (BP) drug class. Successful treatment of osteoporosis using BPs is attributed to their structural similarity to endogenous inorganic pyrophosphates, resulting in their ability to inhibit osteoclast-mediated bone resorption [6,7,8,9,10].

Due to ionisation at physiological pH, orally administered BPs demonstrate extremely low bioavailability (<1%), owing to poor permeability and poor absorption from the gastrointestinal tract (GIT) [11]. Oral BP administration is also sometimes accompanied by local GIT adverse effects, including epigastric pain, dyspepsia and oesophageal ulceration, arising from mucosal irritation [12]. In order to maximise bioavailability, current recommendations are that oral BPs should be taken in the fasted state, at least 30 min prior to the first meal of the day [13]. Physical contact between the oesophagus and BP can be minimised by administering the tablet with a full glass of water and maintaining an upright position for 30 min following ingestion [12]. However, these complex administration conditions can contribute to noncompliance in patients taking oral BPs.

Several approaches have been explored to improve the bioavailability of BPs, minimise their adverse effects and provide long-term drug release. These include the development of BP prodrugs [14,15,16,17,18], BP-loaded hydrogels [19] and encapsulation of BPs into liposomes [20]. However, these systems have not been designed for implantation, and to date there remains no commercially available systems that can provide site-specific, sustained BP release.

Drug-eluting implants (DEIs) are an alternate route of administration with the potential to achieve localised, controlled drug release. Of particular interest are biodegradable DEIs, which use polymers that degrade into biocompatible products that are eventually absorbed or excreted by the body, and hence do not require surgical removal [21]. Two of the most commonly investigated bioerodible polymers include poly(lactic acid) (PLA) and poly(D,L-lactide-*co*-glycolide) (PLGA). In particular, PLGA has been widely used in sustained release formulations as the degradation rate of the polymer can be tailored by controlling the ratio of lactide (LA) and glycolide (GL) units, resulting in tuneable drug release, generally over a period of months to years [22,23,24,25,26,27]. Polymer degradation rate generally increases with increasing GL composition, as GL is more hydrolytically labile than LA [27]. Biodegradable PLA and PLGA implants have previously been used to provide sustained and controlled release of various drugs, including dexamethasone [23], octreotide [28] and gentamycin [29], for use in post-operative cataract inflammation, peptide delivery and chronic osteomyelitis, respectively. Recently, Yao et al. developed an implantable PLA film loaded with tetrandrine for use in reducing scar ingrowths associated with spinal surgery [30]. Release of tetrandrine was achieved through film degradation and drug diffusion, resulting in continuous in vitro drug release over the span of 66 days [30]. Further, Vannozzi et al. reported the fabrication of implantable ultra-thin, multi-layered PLA-polyelectrolyte films loaded with barium titanate nanoparticles and an anti-restenotic drug. These films were found to result in sustained drug release in vitro, which could be controlled using ultrasound stimulation [31]. This highlights the versatility of implantable PLA and PLGA systems across a wide variety of applications.

Hot-melt extrusion has emerged as an attractive approach for the production of implants due to its cost effectiveness, scalability and simplicity, and has been used to non-covalently incorporate a number of drugs into PLGA matrices [32,33,34].

A BP-loaded polymeric implant has the potential to provide sustained release and local drug delivery, improve bioavailability and reduce adverse effects associated with local irritation of the GIT. Hence, in this study, the effect of polymer composition and BP loading on in vitro drug release from bioerodible matrix implants was investigated to aid in the development of an implantable system for the site-specific and sustained delivery of BPs for osteoporosis treatment. To the best of our knowledge, this study will be the first in the literature to describe the preparation of biodegradable BP-loaded implants for the localised and long-term delivery of BPs.

## 2. Materials and Methods

### 2.1. Instrumentation

Proton nuclear magnetic resonance (^1^H NMR) spectroscopy was performed at 298 K using a Bruker NMR AVANCE III HD 500 spectrometer (Bruker BioSpin, Rheinstetten, Germany) or 500 MHz Agilent NMR spectrometer operating at 500 MHz (Agilent Technologies, Santa Clara, CA, USA). Samples were dissolved in CDCl_3_ using the residual solvent peak as an internal reference (CHCl_3_; δ_H_ 7.26 ppm) [35]. Blending of polymer material was performed using a Sunbeam 800W PB7650 MultiBlender (Sunbeam, Botany, Australia). Hot-melt extrusion was performed using a Filabot EX2 extruder (Filabot, Barre, VT, USA) at 170 °C or custom-built extruder (DCXtruder, Adelaide, Australia) at 70 °C; both fitted with a 1.75 mm diameter nozzle. High performance liquid chromatography (HPLC) was performed using a Shimadzu liquid chromatography system fitted with Shimadzu fluorescence (RF-20A) and photodiode array (SPD-M20A) detectors (Shimadzu Corporation, Kyoto, Japan). Separation was achieved using a Waters μBondapak^TM^ (C_18_) 10 μm, 125 Å, 3.9 × 300 mm column (Waters Corporation, Rydalmere, Australia) and 97:3 1 mM ethylenediaminetetraacetic acid (EDTA):methanol (CH_3_OH) mobile phase at a flow rate of 1 mL/min. Fluorescence was measured at λ_ex_ = 395 nm and λ_em_ = 480 nm. Gel-permeation chromatography (GPC) was performed on a Shimadzu liquid chromatography system with UV-visible (SPD-10A) and differential refractive index (RID-20A) detectors (Shimadzu Corporation, Kyoto, Japan), coupled with Shimadzu GPC-804D, GPC8025D and GPC-80MD columns (Shimadzu Corporation, Kyoto, Japan) in series. Tetrahydrofuran was used as the mobile phase at a flow rate of 1 mL/min. Sample concentrations were ~5 mg/mL and were filtered through a 0.45 μM nylon syringe filter prior to analysis. Molecular weight characteristics were determined using a conventional column calibration with poly(ethylene glycol) (PEG) standards. Thermal properties of the polymers were determined using a TA Instruments Discovery differential scanning calorimeter (DSC) (TA Instruments, New Castle, DE, USA). Samples weighing ~2 mg were sealed in aluminium pans and were heated between −80 °C and 200 °C at a ramp rate of 10 °C/min. Thermal transition temperatures were calculated using TA Instruments Universal Analysis (UA) software (TA Instruments, New Castle, DE, USA).

### 2.2. Materials

3,6-Dimethyl-1,4-dioxane-2,5-dione (lactide, 95%) and 1,4-dioxane-2,5-dione (glycolide, 97%) were purchased from BLDpharm (BLD Pharmatech Ltd., Shanghai, China). Tin (II) 2-ethylhexanoate (92.5–100%), tartrazine trisodium salt (95%), anhydrous dioxane and CDCl_3_ were obtained from Sigma-Aldrich, St. Louis, MO, USA. Alendronate sodium trihydrate (ALN·3H_2_O), fluorescamine (>98%) and EDTA were obtained from Carbosynth Ltd., Berkshire, United Kingdom. Methanol, dichloromethane (DCM), tetrahydrofuran (THF), acetonitrile (ACN) and 1-octanol were purchased from ChemSupply, Gillman, Australia. GPC PEG standards were purchased from PSS Polymer Standards Service GmbH, Mainz, Germany. Natureworks 4043D PLA pellets (*M_w_*_(GPC)_ = 200 kDa) were purchased from Filabot, Barre, VT, USA. All chemicals were used as received unless otherwise specified.

### 2.3. Methods

#### 2.3.1. Synthesis of 8:2 PLGA

To a solution of 1-octanol (392 µL, 2.50 mmol) pre-heated at 130 °C was added lactide (43.8 g, 289 mmol) and glycolide (8.64 g, 72.2 mmol) under an atmosphere of argon. After the monomers melted, anhydrous dioxane (20 mL) and tin(II) 2-ethylhexanoate (55 µL, 0.17 mmol, 0.1% *w*/*w*, relative to total mass of lactide and glycolide monomers) were added sequentially, the temperature was increased to 150 °C and the mixture was stirred for 32 h. After cooling to ambient temperature, the crude reaction mixture was dissolved in DCM (200 mL) and then concentrated under reduced pressure to afford the PLGA as a white powder, 48.6 g (97%). ^1^H NMR (500 MHz, CDCl_3_) δ_H_ 0.86 (t, CH_3_, end group), 1.19–1.34 (m, (CH_2_)_4_, end group), 1.44–1.48 (m, (CH_2_)_2_, end group), 1.58 (m, CH_3_, lactide RU), 4.6–4.9 (m, CH_2_, glycolide RU), 5.1–5.3 (m, CH, lactide RU) ppm; NMR end-group analysis: *M_n_* = 8.6 kDa; GPC: *M_w_* = 14.4 kDa, *M_n_* = 8.3 kDa, *Ð* = 1.73.

#### 2.3.2. Synthesis of 9:1 PLGA

To a solution of 1-octanol (196 µL, 1.25 mmol) preheated at 130 °C was added lactide (24.2 g, 159 mmol) and glycolide (2.12 g, 17.7 mmol) under an atmosphere of argon. After the monomers melted, anhydrous dioxane (6 mL) and tin(II) 2-ethylhexanoate (19 µL, 0.059 mmol, 0.1% *w*/*w*, relative to total mass of lactide and glycolide monomers) were added sequentially, the temperature was increased to 150 °C and the mixture was stirred for 5 h. After cooling to ambient temperature, the crude reaction mixture was dissolved in DCM (200 mL) and then concentrated under reduced pressure to give the PLGA as a white powder, 23.8 g (95%). ^1^H NMR (500 MHz, CDCl_3_) δ_H_ 0.89 (t, CH_3_, end group), 1.26–1.32 (m, (CH_2_)_4_, end group), 1.40–1.51 (m, (CH_2_)_2_, end group), 1.55 (m, CH_3_, lactide RU), 4.6–4.9 (m, CH_2_, glycolide RU), 5.1–5.3 (m, CH, lactide RU) ppm; NMR end-group analysis: *M_n_* = 7.1 kDa; GPC: *M_w_* = 10.1 kDa, *M_n_* = 6.1 kDa, *Ð* = 1.68.

#### 2.3.3. Preparation of ALN-Loaded PLGA Implants

PLGA and ALN·3H_2_O were separately ground into a fine powder using a mortar and pestle. High and low drug loadings were achieved by mixing ALN·3H_2_O into the ground PLGA in concentrations of 5% or 18% *w*/*w* (equivalent to 3.9% and 13.9% *w*/*w* alendronate sodium (ALN), respectively). Tartrazine dye was finely ground using a mortar and pestle and incorporated at a concentration of 0.2% *w*/*w* into the PLGA-ALN powder, where specified. The PLGA-ALN powders were then extruded at 70 °C using a 1.75 mm diameter nozzle and the resulting ALN-loaded filaments were cut into rods of 10 mm ± 0.1 mm in length.

#### 2.3.4. Preparation of ALN-Loaded PLA Implants

For low-ALN loadings, PLA pellets (20 g) were dissolved in DCM (100 mL) and precipitated in diethyl ether (1 L). The polymer was isolated by vacuum filtration, followed by blending of the polymer using a Sunbeam 800W PB7650 MultiBlender at 11,000 RPM to obtain a powder. Finely ground ALN·3H_2_O in a concentration of 5% *w*/*w* (equivalent to 3.9% *w*/*w* ALN) was incrementally mixed into the PLA powder. The powder was extruded at 170 °C using a 1.75 mm diameter nozzle and the resulting ALN-loaded filament was cut into implants of 10 mm ± 0.1 mm in length.

For high ALN-loadings, PLA pellets (20 g) were dissolved in DCM (200 mL) to afford a 10% *w*/*v* solution. Finely ground ALN·3H_2_O powder was added to the PLA solution at a concentration of 15% *w*/*w* (equivalent to 11.6% *w*/*w* ALN) and stirred for 30 min. The suspension was poured into a large petri dish (H 25 mm × D 185 mm) and the DCM was evaporated in a fume hood under ambient conditions to obtain an ALN-loaded PLA film that was cut into small pieces (~10 mm × 10 mm) for extrusion. The pieces were extruded at 170 °C with a 1.75 mm diameter nozzle and the resulting ALN-loaded filament was cut into implants of 10 mm ± 0.1 mm in length. Homogeneity following extrusion was visualised through inclusion of tartrazine dye (0.2% *w*/*w*) to the feedstock material, where specified.

#### 2.3.5. Determination of ALN-Loading in PLA and PLGA Implants

The mass of each implant (n = 5 for each formulation) was recorded and the polymeric components of the implants were dissolved in DCM (3 mL). ALN was then extracted from the organic phase with PBS (pH 7.4, 3 mL) and an aliquot of the PBS (100 μL) was used for HPLC quantification.

#### 2.3.6. In Vitro Drug Release Studies from ALN-Loaded PLA and PLGA Implants

Pre-weighed implants (n = 5 for each formulation) were immersed in PBS (pH 7.4, 3 mL) in sealed glass vials and incubated at 37 °C. Aliquots of release media (100 μL) were sampled at regular intervals and replaced with fresh PBS (100 μL). The ALN concentrations in the release media were quantified via HPLC.

#### 2.3.7. HPLC Quantification of ALN Loading and Release from the Implants

ALN concentrations were quantified via HPLC following derivatisation and fluorescence detection (λ_ex_ = 395 nm, λ_em_ = 480 nm), as previously reported [25]. Aliquots of extracts or release media (100 μL) were transferred into a glass vial (4 mL), followed by the addition of 0.13 M EDTA buffer (pH 10, 900 μL) and 2 mg/mL fluorescamine in ACN (500 μL). The solution was then shaken until a yellow colour change was observed (~1 min). DCM (1 mL) was added to the vial and shaken to facilitate removal of unbound fluorescamine and ACN. Once the layers had separated, the upper yellow aqueous layer was withdrawn, centrifuged at 15,000 rpm for 5 min to remove particulate matter and transferred into a glass vial for HPLC analysis. Standard solutions of ALN ranging from 1–250 μg/mL were prepared by serial dilution and subjected to the same derivatisation process as describe above.

## 3. Results and Discussion

### 3.1. Synthesis and Characterisation of PLA and PLGA

Polymers of varying GL content were prepared in order to observe the effect of polymer composition on drug release. Polymers with higher GL content were hypothesised to demonstrate faster rates of release [27,36,37]; an effect ascribed to the more hydrolytically labile nature of the GL repeat units. PLGA copolymers were synthesised via ring-opening polymerisation using LA:GL mole ratios of 8:2 and 9:1 and the drug release profiles from these polymers were compared to that of commercially available PLA. The 8:2 and 9:1 mole ratios were chosen for investigation based on their ideal degradation time in vitro, previously reported to be in the span of 60–100 days [38]. In a clinical context, this prolonged degradation would allow for reduced frequency of implant administration. The exact ratio of LA and GL in the copolymers was determined via ^1^H NMR spectroscopy (Supplementary Information (SI), Figure S1) by comparing the relative integral values for the LA methine and GL methylene proton signals [39]. Using this relationship, the LA:GL ratio in the PLGA copolymers was determined to be 77:23 (~8:2) and 87:13 (~9:1), which is consistent with almost complete conversion of the monomers (97% and 94%, respectively).

The number-average molecular weights (*M_n_*) and dispersity (*Ð*) of the synthesised 8:2 and 9:1 PLGA copolymers as determined by GPC were ~8.3 (*Ð* = 1.73) and 6.1 kDa (*Ð* = 1.68), respectively (SI, Figure S2). These values are in agreement with the *M_n_* values determined from NMR end-group analysis (8.6 and 7.1 kDa, respectively). The relatively high *Ð* values for controlled chain-growth polymerisations were attributed to intramolecular backbiting or intramolecular chain-transfer that commonly occur during prolonged reaction times at high monomer conversions [40]. Such side reactions can be mitigated by terminating polymerisation at a lower monomer conversion; however, polymerisation was run to near completion to negate any extensive purification procedures needed to remove the unreacted monomers.

**Figure 1:**
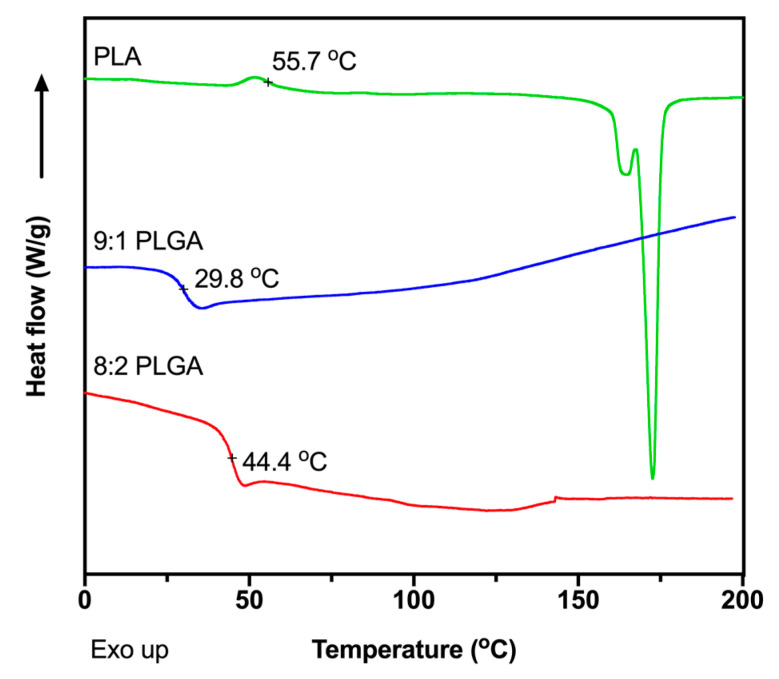
Differential scanning calorimeter (DSC) thermograms showing the second heating cycle for the of 8:2 PLGA, 9:1 PLGA and PLA polymers, recorded at a ramp rate of 10 °C/min.

The glass transition temperatures (*T_g_*) of the polymers were measured using DSC (Figure 1) to determine appropriate extrusion temperatures for preparation of the implants. The *T_g_* values were determined to be 44.4, 29.8 and 55.7 °C for the 8:2 PLGA, 9:1 PLGA and PLA polymers, respectively, consistent with previous reports [39,41,42,43,44]. Also consistent with previous studies, no recognisable melting endotherm was observed in the DSC thermograms of the PLGA copolymers, attributed to the amorphous nature of the polymer [39,44], while the thermogram for PLA demonstrated a melting endotherm at ~175 °C. To facilitate processability, the PLA was extruded at the manufacturer’s recommended processing temperature of 170 °C, while extrusion of the PLGA copolymers was performed ~30 °C above the *T_g_* of the polymer, at 70 °C. Thermal analysis of ALN performed by Albu et al. suggests that, excluding the loss of water, degradation of the BP does not occur until ~260 °C, well above the extrusion temperatures investigated in this study [45]. Extrusion above the *T_g_* of the polymer and below the degradation temperature of ALN would result in the formation of a polymer matrix embedded with solid drug.

### 3.2. Preparation of ALN-Loaded PLA/PLGA Implants

**Figure 2:**
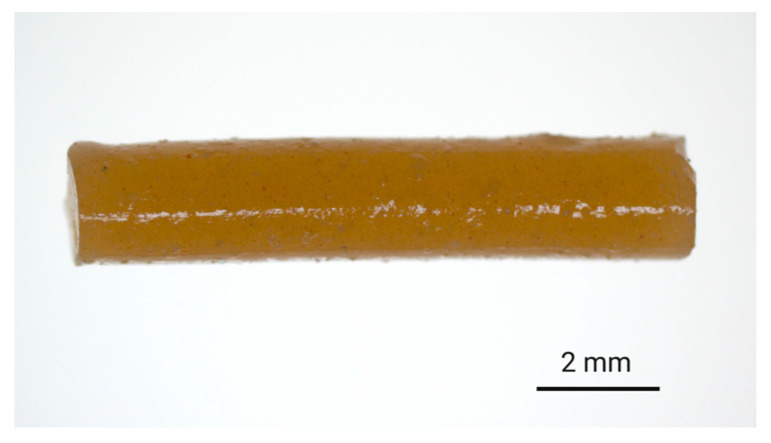
Digital image of the 8:2-PLGA-3% implant (ALN 3% *w*/*w*) containing tartrazine dye (0.2% *w*/*w*), incorporated to provide a visual indication of the distribution of additives (i.e., dye and drug) within the polymer matrix.

ALN-loaded implants were prepared via hot-melt extrusion of ALN-polymer mixtures. For PLGA-based implants, finely ground ALN and PLGA were mixed together in the desired ratio and extruded to afford filament with ALN-loadings of either 3% or 13% *w*/*w*. Subsequently, the filament was cut to provide implants of 10 mm in length. To ensure the homogeneity of the extrudates, a small quantity of hydrophilic tartrazine dye (0.2% *w*/*w*) was added to the feedstock material, which resulted in the formation of evenly coloured filament suggestive of homogenous distribution during the extrusion process (Figure 2).

The addition of a hydrophilic dye was more closely expected to mimic the dispersion of the polar ALN throughout the polymer matrix as compared to a hydrophobic dye. Aside from offering an indication of homogeneity, the dye also served as a visual representation of diffusion from the implant into the release media. The high aqueous solubility of tartrazine ensured that diffusion of the dye from the implant occurred freely and was not limited by solubility. There is a possibility that the addition of tartrazine could contribute to an elevated polymer porosity following dissolution or diffusion of the particles from the implant, potentially influencing the release kinetics. However, due to the low concentration of tartrazine relative to the total mass of the implant (0.2% *w*/*w*), as well as the 15- to 65- fold higher concentration of ALN, any impact of the dye on drug release mechanisms was thought to be negligible. Additionally, the absence of primary amine groups in the chemical structure of tartrazine prevented interference with the derivatisation procedure required for HPLC analysis of the ALN.

For PLA-based implants with an ALN loading of 3% *w*/*w*, PLA pellets were dissolved in DCM, precipitated into diethyl ether and the precipitate was blended into a fine powder prior to mixing with finely ground ALN powder, where the resulting mixture was extruded directly. PLA-based implants with an ALN loading of 10% *w*/*w* were prepared by dissolving PLA pellets in DCM and then adding finely ground ALN to form a suspension. The solvent was then evaporated to provide an ALN-loaded PLA film that was cut into small pieces for extrusion. Following extrusion of the ALN-loaded polymer, the filaments were cut into 10 mm length implants and their mass and diameter were measured in order to determine the dimensional consistency between formulations (Table 1).

With the exception of 8:2-PLGA-3%, the average dimensional measurements (mass, diameter and length) of the implants were reasonably consistent across all formulations. In comparison, the average diameter of the 8:2-PLGA-3% implants were ~0.5 mm greater than the remaining formulations (1.5 mm vs. 2.0 mm, respectively), despite all filaments being extruded using the same nozzle diameter (1.75 mm), which can be attributed to slight variations in extrusion temperature and speed, as at a lower extrusion temperature or slower extrusion speed, the extrudate diameter was observed to increase slightly.

### 3.3. ALN Content Determination in PLA/PLGA Implants

Drug loading has the potential to alter release kinetics from monolithic polymer implants, demonstrated extensively in a number of earlier studies [46,47,48]. Ramchandani and Robinson reported significant differences in the release profile of ciprofloxacin hydrochloride from 50:50 PLGA implants with low (10%–20% *w*/*w*) and high (35%–50% *w*/*w*) drug loadings (changing from biphasic to monophasic release, respectively) [49]. A study by Kunou et al. showed that the release of ganciclovir from 75:25 PLGA implants at 10% drug loading was significantly prolonged compared to drug loadings of 40% (90 vs. 7 days, respectively) [50]. The accelerated release often mediated by higher drug loadings can be attributed to the dissolution of the drug at the polymer surface, triggering pore formation and promoting fluid uptake into the polymer [51]. The subsequent increase in the porosity of the implant enhances the polymer’s surface area and interaction with water, which can encourage further drug dissolution and initiate bulk erosion [51]. High drug loadings may also increase the concentration gradient between the polymer matrix and surrounding media, increasing the rate of drug diffusion from the implant. However, it is important to note that drug loading can also have minimal effect on release kinetics from polymer implants, as reported by Ravivarapu, Moyer and Dunn [52]. Therefore, to investigate the effect of ALN-loading on release from PLA and PLGA, the prepared implants were considered for analysis within two categories: those implants prepared at low ALN-loadings (~3%) and those with high ALN-loadings (~10–13%), summarised in Table 2. A total drug extraction and quantification via HPLC was performed on five samples of each implant type to determine the average ALN-loading (% *w*/*w*) and content uniformity between implants (Table 2).

The average ALN-loadings estimated from the total drug extractions were consistently lower than the loaded quantity of ALN, suggestive of drug loss during the extrusion process, potentially due to drug adsorption to the inside of the barrel, or incomplete extraction of the drug from the implants. In future, additional aqueous extractions could be performed in order to ensure complete extraction of residual ALN from the organic phase. In general, the extrudates were seemingly homogenous, as indicated by the low standard deviations in ALN-loading observed between implants of the same formulation. The comparatively higher variability in ALN-loading for the 9:1-PLGA-13% implants indicates more uneven drug distribution throughout the extrudate, which may have occurred as the ALN approached its solubility limit within the polymer, leading to the formation of phase-separated domains of ALN throughout the length of the extrudate that could subsequently reduce drug uniformity between these implants [53].

### 3.4. Drug Release Studies from ALN-Loaded PLA/PLGA Implants

**Figure 3:**
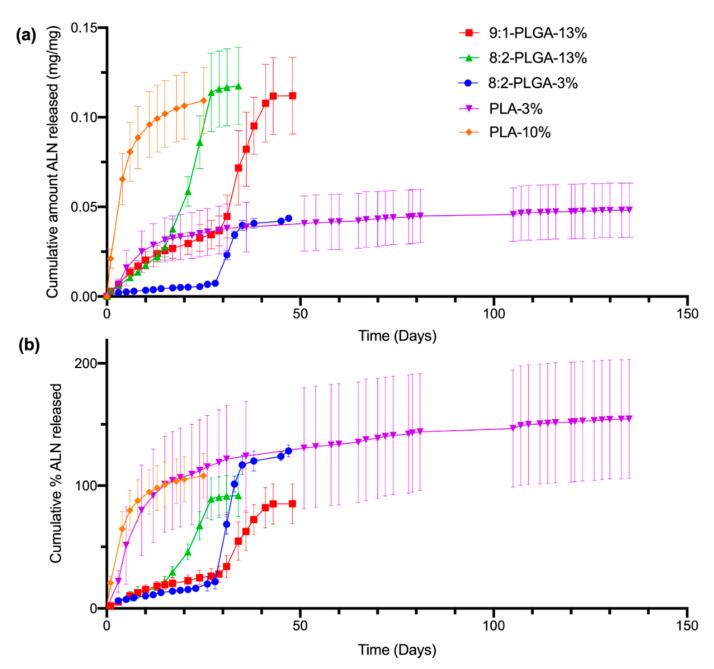
Comparison of the in vitro cumulative amount (mg/mg) of ALN released (**a**) and cumulative % of ALN released (**b**) from all investigated implant formulations in PBS (pH 7.4) at 37 °C. The poly(D,L-lactide-*co*-glycolide) (PLGA) and poly(lactic acid) (PLA) implants demonstrate sigmoidal and logarithmic release profiles, respectively. The total ALN-loading of the implants was calculated from the values listed in Table 2, resulting in percent values that appear to exceed, or plateau below, 100% (for further discussion, see Section 3.4). The values shown represent mean ± SD of n = 4 for 8:2-PLGA-13% and n = 5 for the remaining implant formulations.

The release of ALN from the implants was studied using PBS (pH 7.4) as the release medium under sink conditions at 37 °C and sampling at regular intervals, followed by quantification via HPLC. Sink conditions require a volume of dissolution medium at least 3–10 times the saturation volume of the active pharmaceutical ingredient [54]. This ensures that the rate of drug release is not significantly influenced by saturation of the release media, which can slow the apparent dissolution rate of the drug. The total mass (i.e., 100 %) of ALN within a given implant was based on the average ALN-loading determined from earlier drug extractions (Table 2). The amount of ALN released per sampling period (mg) was calculated from the concentration of ALN found in the PBS release media (μg/mL). Cumulative release was then calculated as the sum of the ALN released from day zero until the corresponding day. Release profiles were obtained by plotting the cumulative amount (mg/mg) (Figure 3a) or cumulative percent (%) (Figure 3b) of ALN released over time.

As evidenced by Figure 3b, the ALN-loadings of the implants investigated in the release study appear to deviate from the predicted-ALN loadings estimated from earlier drug extractions (Table 2), resulting in release profiles that plateau between approximately 70% (9:1-PLGA-13%) to 200% (PLA-3%). While some small variation in ALN-loading may be expected due to the nature of the heterogenous dispersion, there is significant variation in ALN-loading between implants of the PLA-3% implant formulation, despite the low standard deviation obtained in earlier total drug extractions. This discrepancy may be attributed to inefficient incorporation or phase separation of the ALN within the polymer matrix, resulting in implants of high ALN-loading variability [53]. Additionally, incomplete extraction of ALN during the total drug extraction stage could also result in lower calculated estimates of ALN-loading, which may further contribute to this high apparent percentage.

**Figure 4:**
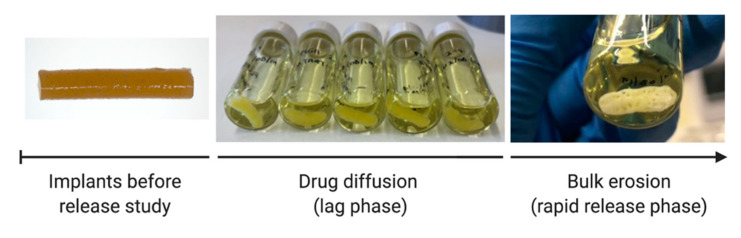
Structural changes to the 8:2-PLGA-3% implants throughout the release study, characterised by initial polymer swelling, followed by macroscopic pore formation at the onset of the bulk erosion period. Diffusion of tartrazine dye (0.2% *w*/*w*) from the implants caused the release media to appear yellow in colour.

All investigated PLGA implant formulations demonstrate sigmoidal release kinetics, characterised by a short initial surface dissolution phase, then a diffusional lag phase, followed by a period of accelerated release. The shape of these release profiles is consistent with those previously reported for PLGA implants loaded with risperidone and ciprofloxacin [49,55]. The initial surface dissolution phase (up to 3 days for 8:2-PLGA-3% and 8:2-PLGA-13% and up to 10 days for 9:1-PLGA-13%) is thought to be caused by rapid dissolution of ALN near the surface of the implants. Following this period, during the diffusional lag phase, ALN is then thought to be released into the surrounding media predominantly by water uptake and diffusion (Figure 4).

The diffusion of tartrazine dye into the PBS, coupled with a decrease in the intensity of the yellow colour at the implant periphery, provided further visual confirmation of water uptake and diffusion processes. As the rate of drug diffusion is much faster than the onset of polymer erosion, it is unlikely that significant bulk erosion took place during this period. Data from the diffusional lag phases of each PLGA implant formulation (8:2-PLGA-3%, 9:1-PLGA-13% and 8:2-PLGA-13%) had good fits for the zero-order diffusion model (R^2^ = 0.97, 0.99, 0.99) (SI, Figure S3), further supporting that ALN release during this period was mediated predominantly by diffusion. Following the lag phase (approximately 14 days for 8:2-PLGA-13% and 28 days for both 8:2-PLGA-3% and 9:1-PLGA-13%), polymer erosion and deformation is then thought to be the predominant mechanism, leading to the observed accelerated drug release from the implants. Dissolution of ALN during the lag phase can form pores within the PLGA matrix that further expedites water uptake, in turn causing hydrolysis of the polymer backbone and resulting in bulk erosion of the polymer and rapid release of encapsulated ALN. However, it is important to note that drug release during this rapid release phase is likely a complex combination of diffusion, bulk erosion and surface erosion.

At the onset of bulk erosion, macroscopic pores appeared throughout the PLGA implants (Figure 4), possibly attributed to differences in the erosion rate within the polymer, where complete hydrolysis of lower *M_w_* chains occurs more rapidly [56,57]. Erosion during this period is also autocatalysed by hydrolysis of ester bonds of the polymer backbone, resulting in the generation of free carboxylic acid groups that can further enhance polymer erosion [27].

Contrastingly, the release profiles for the implants formulated from PLA (PLA-3% and PLA-10%) demonstrated a reduction in ALN release rate over time. This characteristic logarithmic release profile is caused by dissolution of ALN at the surface of the implants, resulting in an increase in thickness of the drug-depleted zone [46]. In turn, this leads to an increased diffusional distance and slower ALN release over time. As the degradation rate for PLA is slower than that for PLGA (due to the absence of GL), no erosion-mediated release phase is observed. Previously reported PLA implants have demonstrated release profiles of similar logarithmic shape [58,59].

In general, implants with higher ALN-loading (10–13%) appeared to demonstrate a faster rate of release compared to implants of the same polymer composition with lower ALN-loadings (3%). This is evident when comparing ALN release from 8:2-PLGA-3% and 8:2-PLGA-13% (Figure 3), where 8:2-PLGA-13% achieves complete drug release after ~27 days, compared to 47 days for 8:2-PLGA-3%. This trend is also apparent on comparison of PLA-3% and PLA-10%. This trend may be attributed to an increase in the ALN concentration gradient between the implant and surrounding release media, leading to accelerated drug diffusion in implants of higher ALN content. In the case of the PLGA implants, accelerated drug diffusion also hastens the rate of pore formation and water uptake into the polymer, leading to a faster onset of hydrolysis-mediated erosion, further reducing the time observed for complete drug release.

**Figure 5:**
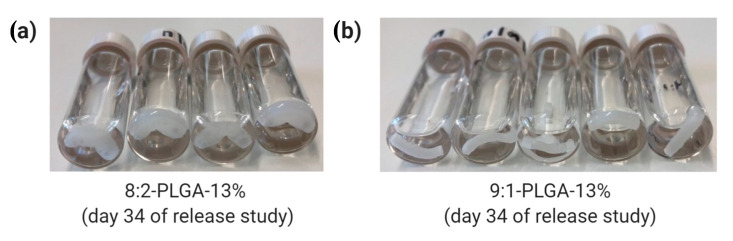
Comparison of water uptake by (**a**) 8:2 and (**b**) 9:1 PLGA implants on day 34 of the release study, where the former exhibits a higher degree of swelling due to higher GL composition.

Through comparison of 8:2-PLGA-13% and 9:1-PLGA-13% it is also evident that the release rate from PLGA implants increases with increasing GL composition, consistent with previous reports [36,37,60]. Due to the absence of a methyl group, GL is more susceptible to hydrolysis than LA, leading to a faster degradation rate in polymers with higher GL composition. Hence, the lag time for 9:1-PLGA-13% is significantly longer than 8:2-PLGA-13% (15 days vs. 29 days) due to prolonged onset of aqueous hydrolysis. Further, the 8:2 PLGA implants (Figure 5a) visibly demonstrated more swelling and deformation compared to the 9:1 PLGA implants (Figure 5b) throughout the study as a result of increased water uptake.

While the PLA-10% implant would be expected to demonstrate prolonged ALN release compared to PLGA implants of similar loading (due to the lack of GL units in the polymer), this formulation released approximately 60% of the incorporated ALN in the first four days. This rapid burst release is thought to be caused by inefficient incorporation of the ALN into the polymer, resulting in rapid dissolution of surface-ALN and the subsequent formation of an extensive porous network that facilitates rapid release of the remaining drug from the implant through dissolution. Similar burst release characteristics have previously been reported at high drug loadings [50].

In the context of therapeutic clinical application, the sigmoidal release profile demonstrated by the PLGA implants may be problematic for long-term drug delivery due to the potential for rapid release and associated systemic toxicity; though this could also be advantageous in scenarios where an increase in dose is desired after a certain time period. Alterations to the polymer *M_w_* and composition could be further explored to accelerate or delay the onset of polymer erosion. Modifying these parameters may produce a viable release profile suitably tailored to a desirable therapeutic release profile of ALN.

## 4. Conclusions

Bioerodible PLGA and PLA implants loaded with ALN were prepared via hot melt extrusion and exhibited sustained drug release between ~25 to 130 days as determined by high-performance liquid chromatography. Release of *bis*phosphonates from these implants was modulated through alterations in the polymer LA:GL ratio, as well as the ALN loading. The PLGA implants demonstrated sigmoidal release profiles as a result of competing mechanisms of drug release, specifically diffusion and polymer erosion. PLA implants demonstrated a logarithmic release profile, characterised by reduced drug release over time, due to increases in the diffusional distance within the implant. Overall, these implants represent a promising initial platform from which sustained-release *bis*phosphonate implants may one-day be developed in an effort to overcome the administration issues associated with oral *bis*phosphonates.

## Figures and Tables

**Table 1:** Average dimensional measurements of the investigated alendronate sodium (ALN)-loaded implant formulations prepared by hot-melt extrusion.

Implant Formulation ^a^	Average Mass ± SD (mg) ^b^	Average Diameter ± SD (mm) ^b^	Average Length ± SD (mm) ^b^
8:2-PLGA-3%	41.6 ± 1.7	1.99 ± 0.05	10.05 ± 0.04
PLA-3%	23.0 ± 0.9	1.54 ± 0.02	9.96 ± 0.1
PLA-10%	24.6 ± 0.4	1.54 ± 0.02	10.07 ± 0.03
9:1-PLGA-13%	24.5 ± 3.0	1.54 ± 0.1	10.09 ± 0.1
8:2-PLGA-13%	24.5 ± 2.9	1.45 ± 0.2	10.06 ± 0.05

^a^ Implant codes represent LA:GL ratio-polymer-ALN loading (% *w*/*w*), whereby the ALN-loading was determined experimentally via high performance liquid chromatography (HPLC). ^b^ Averages and standard deviations calculated from n = 5.

**Table 2:** Summary of investigated ALN-loaded implant formulations and their average ALN-loadings determined from total drug extractions.

Implant Code ^a^	Polymer	LA:GL Ratio	ALN Added (% *w*/*w*)	ALN-Loading (% *w*/*w* ± SD) (n = 5)
8:2-PLGA-3%	PLGA	8:2	3.9	3.4 ± 0.6
PLA-3%	PLA	10:0	3.9	3.1 ± 0.6
PLA-10%	PLA	10:0	11.6	10.2 ± 1.3
9:1-PLGA-13%	PLGA	9:1	13.9	13.1 ± 2.6
8:2-PLGA-13%	PLGA	8:2	13.9	12.8 ± 0.6

^a^ Implant codes represent lactide (LA):glycolide (GL) ratio-polymer-ALN loading (% *w*/*w*), whereby the ALN-loading was determined experimentally via HPLC.

## Supplementary Materials

The following are available online at https://www.mdpi.com/2073-4360/12/12/2930/s1, Figure S1: ^1^H NMR spectra of 8:2 and 9:1 PLGA, Figure S2: GPC chromatograms of PLA, 8:2 PLGA and 9:1 PLGA, Figure S3: Data from diffusional lag periods of PLGA implants fitted to the zero-order release model.
