# Supplementary material for: Drug-Eluting Biodegradable Implants for the Sustained Release of Bisphosphonates
Source: Polymers (Basel). 2020 Dec 7;12(12):2930. doi: 10.3390/polym12122930 (PMC7762379; doi:10.3390/polym12122930)
Supplement: Supplementary file 1 [file polymers-12-02930-s001.pdf]

Supplementary Materials: The following are available online at www.mdpi.com/xxx/s1, Figure S1: ^{1}H NMR spectra of 8:2 and 9:1 PLGA, Figure S2: GPC chromatograms of PLA, 8:2 PLGA and 9:1 PLGA, Figure S3: Data from diffusional lag periods of PLGA implants fitted to the zero-order release model.

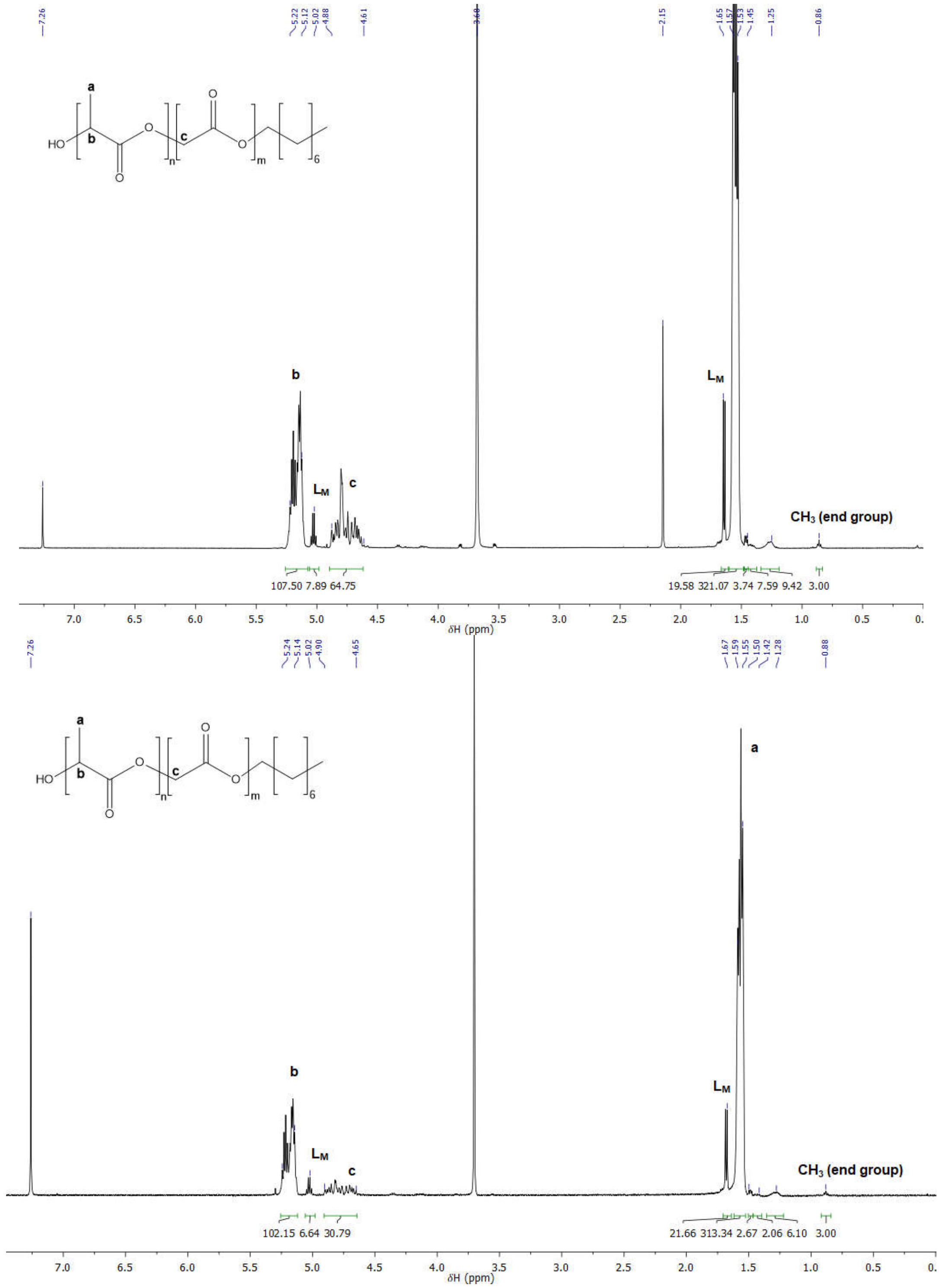

Figure S1. ^{1}H NMR spectra (500 MHz, $CDCl_3$) of synthesized 8:2 PLGA (top) and 9:1 PLGA (bottom). Residual lactide monomer signals are denoted as L_M. The LA:GL ratio in the synthesized polymers was determined using equation (1) below. I_L and I_G correspond to the integration values of the LA methine and GL methylene protons, respectively, while F_G represents the fraction of GL in the copolymer [36].

$$F_G = \frac{I_G}{(2I_L + I_G)} \quad (1)$$

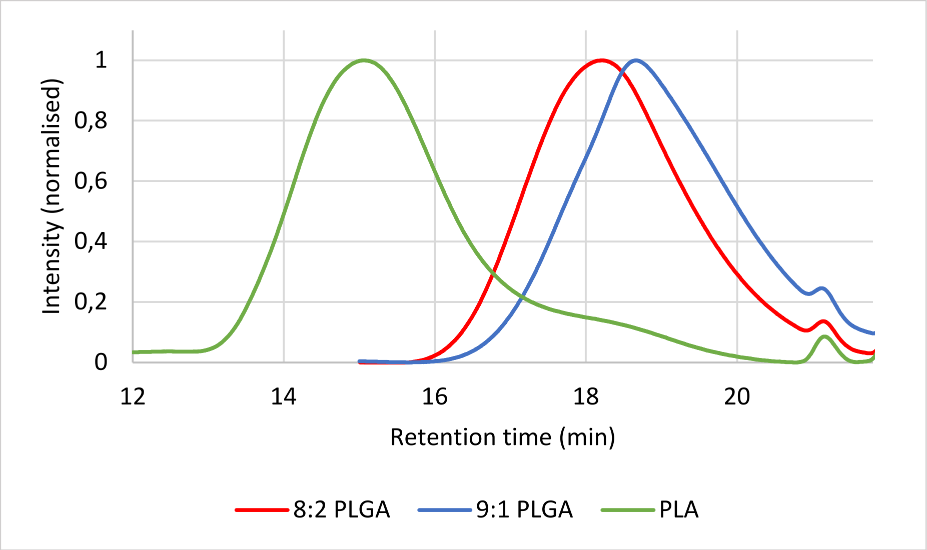

Figure S2. GPC differential refractive index chromatograms of 8:2 PLGA, 9:1 PLGA and PLA.

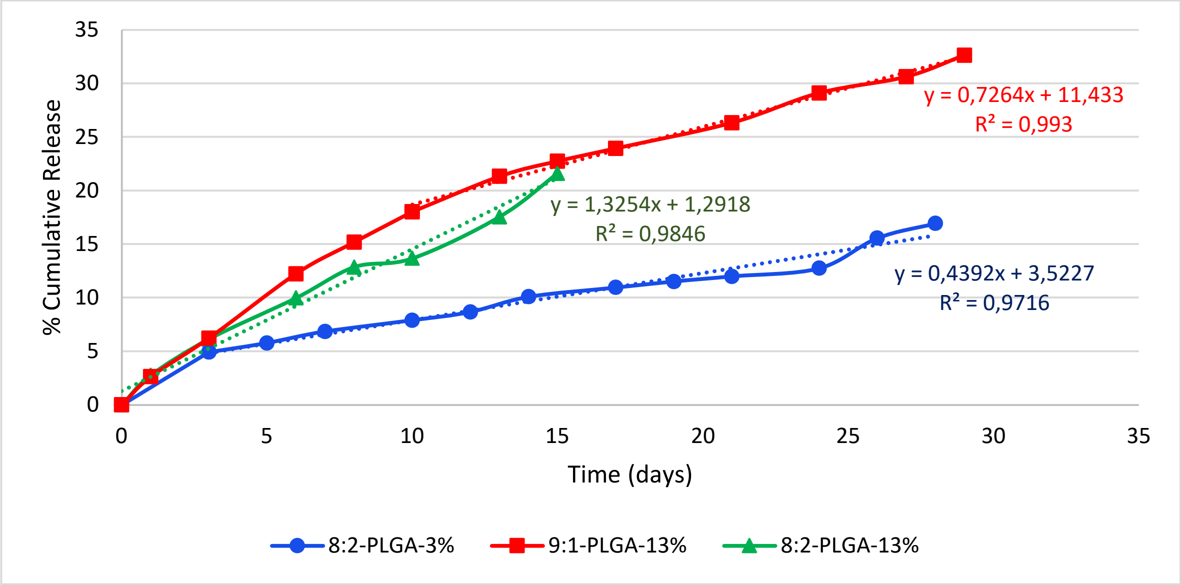

Figure S3. PLGA implants demonstrated good fits for the zero-order diffusion model during their respective diffusional lag periods. The initial rapid release observed prior to the diffusional lag period are attributed to surface dissolution of ALN from the implant and have not been included in the linear regression.
